# Effectiveness of Exercise in Patients with Overweight or Obesity Suffering from Knee Osteoarthritis: A Systematic Review and Meta-Analysis

**DOI:** 10.3390/ijerph191710510

**Published:** 2022-08-24

**Authors:** Jose Manuel Jurado-Castro, Mariano Muñoz-López, Agustín Sánchez-Toledo Ledesma, Antonio Ranchal-Sanchez

**Affiliations:** 1 Metabolism and Investigation Unit, Maimonides Biomedical Research Institute of Cordoba (IMIBIC), Reina Sofia University Hospital, University of Cordoba, 14004 Cordoba, Spain; 2 Ciencias De La Actividad Física y El Deporte, Escuela Universitaria de Osuna (Centro Adscrito a la Universidad de Sevilla), 41640 Osuna, Spain; 3 Instituto de Seguridad y Bienestar Laboral, 14001 Cordoba, Spain; 4 Higher School of Engineering and Technology, International University of La Rioja (UNIR), 26004 Logroño, Spain; 5 Department of Nursing, Pharmacology and Physiotherapy, Faculty of Medicine and Nursing, University of Cordoba, 14071 Cordoba, Spain; 6 Grupo De Investigación Clínico Epidemiológica De Atención Primaria, Maimonides Biomedical Research Institute of Cordoba (IMIBIC), Reina Sofia University Hospital, University of Cordoba, 14004 Cordoba, Spain

**Keywords:** exercise, osteoarthritis, obesity, physical function, fall prevention

## Abstract

Objectives: The main purpose of this study was to review the evidence about the effectiveness of exercise in patients with overweight or obesity suffering from knee osteoarthritis. Methods: Randomized clinical trials (RCTs) published between January 2002 and May 2022 were included. Results: A total of 64 articles were identified, of which six met the criteria for meta-analysis. The pain scale score was higher in the control group (mean difference 0.95; confidence interval 0.42–1.47; *p* < 0.001; I^2^ = 44%). The physical function scale (lower scores indicate lower levels of symptoms or physical disability) presented a higher score in the control group (mean difference 3.74; confidence interval 0.85–6.53; *p* < 0.05; I^2^ = 56%). Moreover, the intervention group achieved a greater distance (meters) walking in a 6 min interval (mean difference 38.18; confidence interval 20.01–56.35; *p* < 0.001; I^2^ = 0%). Conclusions: Exercise interventions seem effective in improving quality of life in people with overweight or obesity suffering from knee osteoarthritis, reducing pain and improving physical function.

## 1. Introduction

Life expectancy has increased globally over time, yet the growing burden of chronic disease means that a large part of society is living longer, but in poorer health. This scenario is a reality for people who suffer from one of the main causes of chronic pain and disability worldwide, such as osteoarthritis [1].

The body areas that can be affected by osteoarthritis are the hands, the cervical and lumbar spine, the hips, and the knees. According to global data, osteoarthritis affected about 3.64% of the world population in 2010 [2]. The knee joint, a key factor in corporal balance, is the most commonly affected lower-limb joint [3]. Knee osteoarthritis (KO) ranks as the 10th largest contributor to global years lived with disability, and its prevalence has more than doubled during the last decade [1]. The average cost of knee and hip osteoarthritis for a developed country, such as Spain, is EUR 4738 million, of which 46% corresponds to healthcare expenses, 22% to sick leave, 13% to hospital admissions, 7% to diagnostic tests, and 5% to drugs [4].

KO affects the entire joint and causes synovial inflammation, cartilage damage, bone remodeling, and osteophyte formation. Typical symptoms include pain, muscle weakness, joint instability, stiffness, crepitus, and functional limitations [1]. Evidence shows that, despite the fact that patients with KO have higher bone mass, they have an increased risk of fractures, which seems to be related to an increase in the number of falls among these patients [5]. In addition, it appears that KO patients have a greater propensity to run into obstacles [6]. It has been observed that the increase in falls observed in patients with osteoarthritis of any location (up to a 27% increase in incident falls) explains the increase of up to 40% in the risk of fracture observed in this population after a prospective follow-up of 3 years [7]. It is known that fractures in old people, especially in the hip, are the beginning of the end for many. Thus, prevention and control of the main risk factors remains essential.

In KO, obesity is one of the most relevant risk factors for this pathology. Mechanical overload on the joints activates an inadequate chondrocyte response that accelerates cartilage degeneration. It has also been described that obesity and overweight have a systemic effect due to the pro-inflammatory and degenerative role attributed to some adipokines secreted by adipose tissue, speeding up the cartilage and bone catabolism independently of the mechanical effect [8]. In addition, the physical condition is limited in people with obesity and KO [9,10,11], and a high body fat percentage (BFP) is usually associated with sarcopenia [12]. Then, people with overweight or obesity suffering from KO usually have disability due to sarcopenia, which favors the propensity to fall given the weakness in the muscles of the lower limbs.

Furthermore, the displacement in the body’s center of gravity in people with obesity has to be considered, since older adults with central obesity are more likely to experience falls [13]. In this sense, a recent study shows that the increase in BFP is a factor that simultaneously caused a decrease in gait performance and an increase in gait instability in older males, and BFP was consequently the most important factor for a stable gait in older males. Thus, given the increase in fall risk when persons with obesity also suffer from KO, it is reasonable to suggest that improving body composition is likely to contribute to reducing fall risk.

Available KO treatments include drug therapies, intra-articular injections, surgical procedures, and conservative interventions such as physical therapy, braces, devices, and physical exercise [1]. Rheumatologists and orthopedic doctors recommend conservative interventions as a fundamental strategy for the management of mild to moderate KO. Often, these conservative approaches must be combined with pharmacological and surgical treatments for optimal management of the disease. All interventions can help to reduce pain associated with KO. An important advantage of nonpharmacologic/nonsurgical approaches, particularly those interventions that include exercise, is their direct effect on improving physical function [14]. Furthermore, physical activity and exercise have been shown to be a central component because it has been shown that a reduction in body weight directly affects a reduction in the load on the joint and, therefore, a reduction in pain and inflammation [1,14]. In addition, this type of intervention seems to be effective in reducing the risk of falls and, therefore, preventing fractures [15].

In order to update the evidence about the themes discussed above, the objective of this study was to review the evidence about the effectiveness of exercise in patients with overweight or obesity suffering from KO through a systematic review and meta-analysis.

## 2. Materials and Methods

A systematic review and meta-analysis were carried out according to the Preferred Reporting Items for Systematic Reviews and Meta-Analysis (PRISMA) [16]. The PRISMA criteria were assessed by a check-list (Supplementary Material File S1).

Furthermore, the revision protocol was registered in the prospective registered systematic reviews in health and social care, international database, PROSPERO, with registry code CRD42022342305.

### 2.1. Criteria for Considering Studies for Inclusion in the Review

Randomized clinical trials (RCTs) published between January 2002 and May 2022 were included in this systematic review and meta-analysis.

Inclusion criteria were established according to the PICOS strategy for the research question. The “Population” (P): patients with overweight or obesity and KO; “Intervention” (I): physical exercise as treatment; “Control” (C): groups of patients with these conditions who did not receive any exercise intervention; “Outcomes” (O): pain, functional capacity measured with the 6 min walk test (6MWT)/Western Ontario and McMaster Universities Osteoarthritis Index (WOMAC) physical function scale; and the “Studies” (S): RCTs.

Non-primary studies (literature review, editorials, book chapters) and other “grey literature” were excluded. Unreadable studies and studies in languages other than English were excluded, in addition to those that did not report the proposed outcomes or whose full text was not available.

### 2.2. Protocol for Electronic Searching

The bibliographic search was carried out through the PubMed, Web of Science (WOS), and SCOPUS electronic databases.

The Medical Subject Headings of “Knee Osteoarthritis”, “Exercise”, and “Obesity” terms were combined with the Boolean operator “AND”. More details about the electronic search are shown in the Supplementary Material (Supplementary Material File S2). All found papers were critically assessed in order to choose those that met the proposed criteria.

### 2.3. Study Selection and Data Collection

Two researchers (J.M.J.-C. and M.M.-L.) conducted the literature search and analyzed the results independently. Then, the articles were codified through the computerized reference manager RefWorks [17], and the discrepancies were discussed regarding the interpretation of the extracted data. The articles were filtered according to the inclusion criteria.

The bibliography search was performed in two phases. During the first phase, papers were selected based on their title and abstract. Those articles that did not meet the inclusion criteria were discarded. In the second phase, all of the selected articles were carefully read and analyzed. Information and data about the characteristics of the population and participant number, type, and duration of treatment exercise intervention, and the main results of the studies, were extracted.

### 2.4. Risk of Bias in Individual Studies

The risk of bias assessment was assessed following the recommendations of the Cochrane Collaboration [18]. For each study, seven domains were scored with high, low, or unclear risk of bias. These domains were: sequence generation, allocation concealment, blinding of participants and staff, blinding of outcome assessment, incomplete outcome data, selective outcome reporting, and other issues considered.

### 2.5. Statistical Analysis

The effect of exercise interventions was analyzed by comparing the experimental group (EG) with a control group (CG) that did not exercise. Data were obtained using the mean difference (MD) and standard deviation (SD) of assessment data (numerical values) shown after the intervention.

The numerical values presented in the selected studies were assigned a value of 0–20 for the pain variable [19] (lower scores indicate lower levels of pain), in order to standardize the results for further analysis and reduce heterogeneity. The physical function variable was standardized according to the WOMAC scale with a value of 0–68 [20] (lower scores indicate lower levels of symptoms or physical disability). The 6MWT variable was quantitative, recording the total distance reached, in meters [21].

The results of this meta-analysis are shown as a “forest-plot” with MD and 95% confidence interval (CI). Heterogeneity is also presented, and was calculated by measuring its extent by the I^2^ index. The *p*-value for this statistic was examined noting the presence of heterogeneity when *p* < 0.05, which compromised the validity of the pooled estimates [22]. Furthermore, the I^2^ index of heterogeneity was considered low when values were between 0% and 40%; moderate between 30% and 60%; considerable between 50% and 90 %; and substantial between 75% and 100% [23]. In addition, since the population and interventions of this study were presumed to be inhomogeneous, a random-effects model was used to measure the effect of the included studies [24].

The Review Manager (RevMan) software 5.4.1. (Cochrane Collaboration, Oxford, UK) was used to perform the meta-analysis [25]. Statistical significance was considered in all analyses with a value *p* < 0.05. The results are shown with the MD followed by the SD.

## 3. Results

### 3.1. Studies Selected

**Figure 1:**
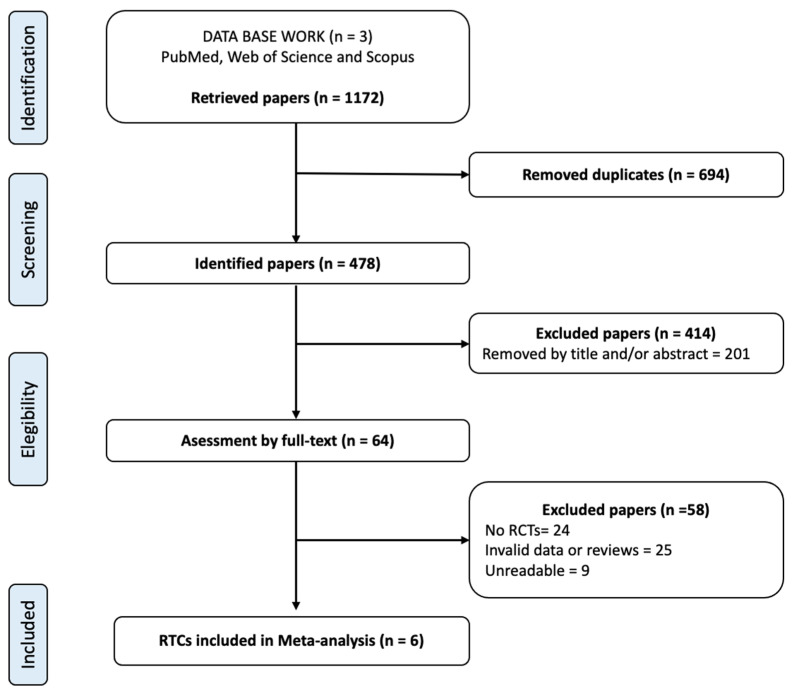
Flow diagram for the scientific paper selection from databases. RCTs, randomized controlled trials.

A flowchart diagram describes the selection of articles that were included in this meta-analysis (Figure 1). A total of 1172 papers were identified from the various included databases following the review. A total of 694 articles were deleted as duplicates, leaving 478 potential papers to be selected for inclusion. A total of 414 articles were eliminated because the title or abstract was not associated with the aims. Following this, 64 full-text articles were considered to be potentially eligible according to the inclusion criteria. However, 58 were eliminated because they did not meet the inclusion criteria or were illegible. Thus, six articles were finally selected for the present meta-analysis.

### 3.2. Description of Selected Studies

The characteristics of the included studies are provided in Table 1. Two of the studies were conducted in the United States [26,27]; one in the Netherlands [28]; one in China [29]; one in Korea [30]; and one in Malaysia [31]. A total of 217 patients (64 ± 5 years old) with overweight or obesity participated in an exercise intervention compared to 193 (63 ± 5 years old) who were recruited to a control group without exercise intervention. Four studies included only patients with obesity in their sample [26,27,28,31], while two studies had a sample made up of patients with overweight or obesity [29,30]. The interventions lasted from 1 month [31] to 18 months [26], with the mean duration of the interventions being 7.57 ± 6 months. Two of the included studies [26,29] incorporated an exercise and diet intervention, in addition to an exercise intervention. These studies had a diet-only group, from which data were selected for the control group in the forest-plots of the meta-analysis.

The six studies included in this meta-analysis included the physical function variable in their results [26,27,28,29,30,31], five studies included the pain variable [26,28,29,30,31], and three studies presented the 6MWT variable [26,27,28].

The Lim et al. study [30] was used twice in the meta-analysis because it incorporated two different exercise conditions (i.e., exercise in water and exercise on land), both compared to a control group.

### 3.3. Risk of Bias in Included Studies

**Figure 2:**
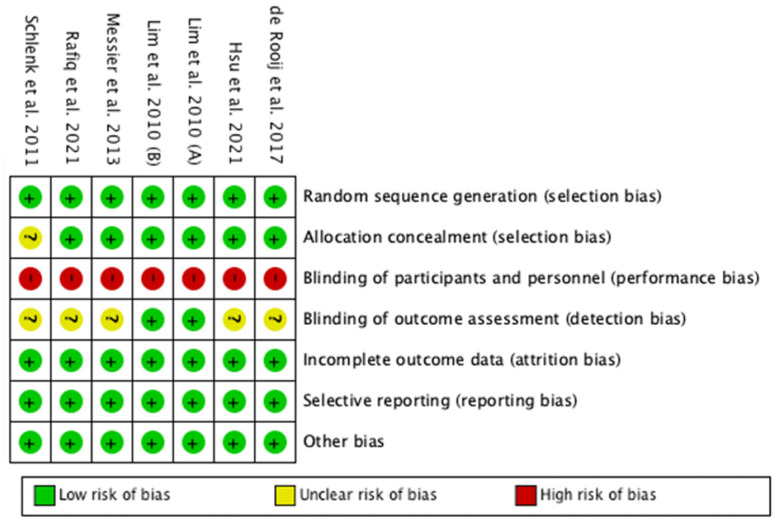
Risk of bias summary: review authors’ judgements about each risk of bias item for each included study ([26,27,28,29,30,31]).

The included studies were based on a physical exercise intervention, in which the participants who carried it out were aware of their participation. Except for this, however, no high risks of bias were detected within the seven domains analyzed for any of the studies included in this meta-analysis (Figure 2).

### 3.4. Effects of the Interventions

#### 3.4.1. Pain

**Figure 3:**
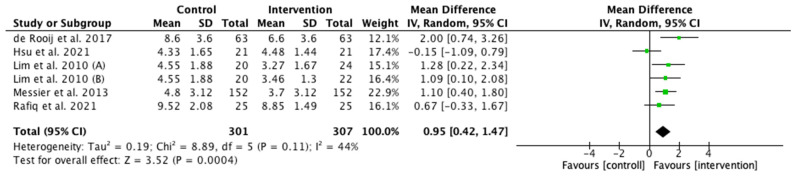
Effects interventions on pain. CI, confidence interval. Note: lower scores indicate lower levels of pain ([26,28,29,30,31]).

The pain scale score was higher in the control group (MD 0.95; CI 0.42–1.47; *p* < 0.001; I^2^ = 44%). Messier et al. 2013 [26] presented the highest weight (22.9%) (Figure 3).

#### 3.4.2. Physical function

**Figure 4:**
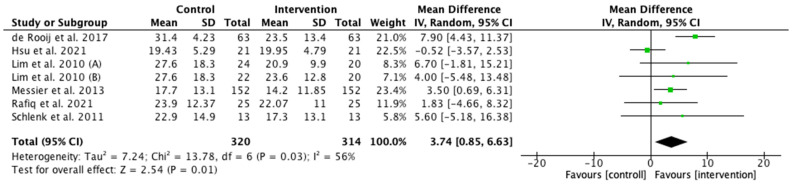
Effects of interventions on physical function. CI, confidence interval. Note: lower scores indicate lower levels of symptoms or physical disability ([26,27,28,29,30,31]).

Physical function scale presented a higher score in the control group (MD 3.74; CI 0.85–6.63; *p* < 0.05; I^2^ = 56%). Messier et al. 2013 [26] presented the highest weight (23.4%) (Figure 4).

#### 3.4.3. Six-Minute Walk Test

**Figure 5:**
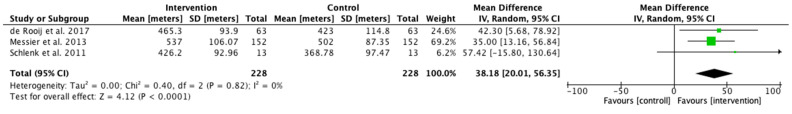
Effects of interventions on distance (meters) reached in the 6 min walk test. CI, confidence interval ([26,27,28]).

The 6MWT showed an increase in favor of the intervention group (MD 38.18; CI 20.01–56.35; *p* < 0.001; I^2^ = 0%). Messier et al. 2013 [26] presented the highest weight (69.2%) (Figure 5).

### 3.5. Sensitivity Analysis

A sensitivity analysis excluding studies [26,29] comparing a “diet and exercise” group versus a “diet” group is presented in Supplementary Material (Supplementary Material File S3). This reduced heterogeneity to 0%, but without altering the results and interpretation of the meta-analysis (Figure 3, Figure 4 and Figure 5).

## 4. Discussion

The objective of this study was to review the evidence about the effectiveness of physical exercise in patients with overweight or obesity suffering from KO, through a systematic review and meta-analysis. The main findings show that there is evidence about the effectiveness of the physical exercise interventions in patients suffering from these comorbidities. The study of Messier et al., 2013 [26] presented the greatest weight in pain relief, physical function improvement, and the 6MWT distance in the patients with the pathologies considered in this study. This RCT showed the positive effects of a program that combines an intensive diet and exercise on knee joint loads, inflammation, and clinical outcomes among adults with overweight and obesity.

Osteoarthritis is a condition that causes joints such as the knee to become painful and stiff, reducing the patients’ daily activity. Hence, improving the musculoskeletal function is relevant. De Rooij et al., 2017 [28] indicated the mean improvements in the intervention group (33% on the WOMAC scale and 15% on the 6MWT) after three months of tailored exercise therapy in a physical functioning program. Moreover, Schlenk et al., 2011 [27] showed significant increases in self-reported performance of lower extremity exercise and participation in fitness walking. All of the analyzed studies [26,27,28] from which the data for the 6MWT were extracted showed an improvement in the distance walked in the intervention group. Consequently, physical activity is a key intervention to increase the individual functional capacity. Recently, another physical test [32] has been proposed; this is the 10 s one-legged stance performance, which predicts survival in middle-aged and older individuals, and may be a part of a routine physical examination, including of KO patients.

Similarly, the literature regarding management of KO [33,34,35,36,37,38] shows greater improvements in pain, function, or both pain and function over the non-exercise control group, although in our work we focused in patients with overweight or obesity also suffering from this pathology. This aspect has not been reviewed by a meta-analysis so far.

One important question about patients who suffer from KO is the practical utility of functional improvement. Since typical KO symptoms include muscle weakness, pain, and functional limitations, the physical function improvement is essential to prevent falls and the consequent serious adverse medical aftermath in KO patients with frailty. It is known that, despite having a higher bone mass, people with KO have an increased risk of fractures, which seems to be due to an increase in the prevalence of falling among these patients [5], and particularly in older persons and postmenopausal women. In this sense, variables such as overweight or obesity, body composition, sarcopenia, and sex have to be considered in patients who suffer from KO.

In regards to age, overweight or obesity, body composition, and ageing are associated with a progressive decline in physical fitness [39], including muscle strength, muscle power, flexibility, balance, and body composition [32]. The knee joint is crucial to corporal balance. Regarding balance and body composition, it is reasonable to consider that the fall risk increases with the increased degree of central obesity in individuals with KO, which makes them more fragile and vulnerable. Lee et al. show in their 2022 study [40] that the increase in the body fat percentage is a factor that simultaneously causes a decrease in gait performance and, on the contrary, an increase in gait instability in older people. As the person is older and they have a higher body weight, they have a higher risk of suffering KO. In fact, Beom-Young et al. [13] mention that older adults with central obesity are more likely to experience a fall compared to with those without central obesity. In the studies included in the conducted meta-analysis, Hsu et al., 2021 [29] conclude that individual diet combined with telemedicine-based resistance exercise intervention significantly improved the body composition, in addition to the blood biochemistry and lower-limb functional performance, of the investigated population with obesity and KO conditions. Nevertheless, Rafiq et al., 2021 [31] mentioned that short-term effects of the lower-limb rehabilitation protocol appear to reduce knee pain and stiffness only, but not physical function or BMI.

Sarcopenia is another important condition to be considered in people with overweight or obesity suffering KO [41]. Different observational studies indicate that older people with KO have lower muscle mass or volume compared to healthy controls [42,43,44,45]. There is also evidence indicating that having less muscle is further associated with lower muscle strength/power and poor functional outcomes [46,47]. Recent studies have reported that KO is associated with a high risk of sarcopenia [48,49], an age-related condition characterized by attenuated muscle mass [50,51]. Age-related changes in muscle mass have been suggested to be associated with decreased strength and walking speed [52,53]. In contrast, intervention-induced programs that increase muscle mass may improve strength and walking ability in older people who have sarcopenia and are at risk of frailty [54]. Furthermore, there is an expert consensus that the combination of sarcopenic obesity and loss of flexibility and balance is harmful to overall health, increasing the fragility of older people and, consequently, the risk of falls [55]. This is an important issue because falls are the second-highest cause of unintentional injury-based deaths worldwide [32]. Moreover, the number of accidents in the geriatric population older than 65 years old is increasing, for which the common cause is a multifunctional gait disorder [56]. Therefore, physical exercise programs followed by patients with KO to increase their lean muscle mass and muscle power would reduce such risks. A recent meta-analysis [57] evaluated the relative efficacy of weight control, exercise, and combined treatment for muscle mass and physical sarcopenia indices in overweight and obese adults having KO. Results demonstrated that exercise strength alone and strength exercise with blood flow restriction are, in general, two optimal treatments for muscle strength gains in people with obesity and KO. This fact may lead to a reduction in the risk of falling in older adults with these problems [58]. In this sense, future studies that include other types of exercise, such as proprioceptive exercises, in addition to factors such as restful sleep and heat stress, will be required.

This meta-analysis compared interventions (physical exercise) and no intervention groups, with or without diet. Two of the included studies [26,29] incorporated an exercise and diet intervention, in addition to an exercise intervention. According to these studies [26,29] that compared the “diet” intervention and “diet plus exercise” intervention, the latter group obtained better results. According to the results, nutritional intervention (diet) combined with physical exercise is a relevant point to consider in the treatment of KO. Sensitivity analysis (Supplementary Material File S3) excluding studies [26,29] that also had diet in their intervention showed a reduction in heterogeneity to 0% (Figures S1–S3), but without altering the results and interpretation of the meta-analysis.

In this sense, as mentioned in the guidelines of the Osteoarthritis Research Society International (OARSI) [59], in the case of overweight, weight loss translates into a lasting reduction in the joint pain it produces. A minimum weight loss of 5% has to be achieved to show a clinically significant effect [60]. In their meta-analysis, Lim et al. [30] indicate that BMI showed a small reduction in the exercise group, even in the water exercise group. However, another prospective cohort study [61] and a systematic review and meta-analysis study [62] showed that, although the groups that followed a “dietary intervention” reported a reduction in BMI, they lost both fat and muscle, whereas in the “diet plus exercise” groups, muscle mass was maintained, leading to improvements in pain and stability by reducing compressive force on joints. Thus, gains in muscle mass caused by exercise may be beneficial for older adults with obesity undergoing obesity weight control.

Regarding the relation between pain and BMI, Van Gool et al. [63] conclude that pain and BMI reduction helped explain, to some extent, the link between exercise adherence and changes in physical performance and self-reported disability among overweight older adults with KO. One key point is to engage with and achieve adherence in an exercise program that best suits the patient. Another factor is pain control, which is certainly a major aspect for KO patients’ quality of life. Meisser et al., [26] conclude that the pain variable decreases in the exercise and diet intervention group versus the non-intervention group (diet only). Lim et al. [30] mention that water exercise reduces the degree of pain via activity interference. In addition, Rafiq et al. [31] show that short-term effects of the lower-limb rehabilitation protocol appear to reduce knee pain and stiffness. Similar to our results, the studies of patients with KO [33,34,35,36,37,38] show greater improvements in pain, function, or both pain and function due to exercise intervention.

By comparison, disability patients with KO and sarcopenia may benefit from exercise programs combined with examined supplements (creatine, for example). In addition to muscle empowerment supplements, others such as turmeric, ginger extract, glucosamine, sulfate chondroitin, and vitamin D may be helpful in reducing pain and improving function in patients with mild to moderate KO, although the evidence is inconsistent [64]. Accordingly, additional research clarifying the efficacy of each supplement is needed, in addition to studies that examine the physical exercise of patients compared to the combination with some of these supplements.

Tailored exercise is a key factor to improve the physical condition in patients with overweight or obesity suffering from KO. De Rooij et al., 2017 [28] indicate that tailored exercise therapy is efficacious in improving physical functioning in patients with KO and severe comorbidities. Another point to be considered is the convenience of exercise prescribed by experts. For example, aquatic exercise is suitable for people with obesity and KO who find it difficult to begin exercises that overburden the knee. The literature shows that supervised exercise, unsupervised exercise, and/or aquatic exercise are recommended over no exercise to improve pain and function treatment of KO, with a “strong recommendation” [64]. Similar to the meta-analysis results, Lim et al. [30] conclude that water exercise may be an effective tool for patients with obesity who have difficulty performing conventional exercises because of the combination of KO. In addition, it may be interesting to note the convenience of coaching people with obesity and KO regarding their daily activities. For example, despite the benefits of physical activity, it may be beneficial for certain patients to walk up stairs, but not to walk down them, for which the use of an elevator would be safer in order to prevent falls.

The knowledge regarding the evidence in successful interventions in this condition and comorbidities may contribute to a better quality of life in these patients. Schlenk et al. conclude that these interventions are feasible and acceptable, and improve physical activity and function [27].

Finally, sex is also a variable to consider. Prieto-Alhama et al. [7] indicate that postmenopausal women with self-reported osteoarthritis have a 20% increased risk of fracture and experience 25% more falls than osteoarthritis-free peers. In addition, in elderly women with KO, knee muscle strength, and especially extensor strength, decreases significantly [44]. In fact, Zhang et al. [45] indicate that, in women, the quadriceps muscle and the rear-thigh muscle (which maintains the stability of knee joints during rehabilitation training) should be strengthened rather than following a program of weight loss alone.

### Limitations

The findings of this study must be interpreted according to a series of limitations. The first of these is the variation in the exercise protocols of the included studies. Secondly, the meta-analysis has the limitation of differentiating the sex variable in the results. Another limitation is the absence of the assessment of risk of bias that may affect the cumulative evidence, and the small number of articles considered for the meta-analysis.

Furthermore, due to the limited number of articles, articles that included diet in their intervention in addition to exercise were selected, which may be a confounding aspect in the results. However, the exercise and diet group was compared to a diet group, rather than a control group without any intervention.

## 5. Conclusions

The analyzed exercise interventions, which were found to be feasible and acceptable, are effective in improving the quality of life in people with overweight or obesity suffering from KO, reducing pain and improving physical function.

The study of Messier et al. [26] presented the greatest weight in pain relief, physical function improvement, and the 6MWT distance in the patients with these comorbidities. This shows the positive effects of a program that combines an intensive diet and exercise incorporating knee joint loads, in terms of inflammation and clinical outcomes, among adults with overweight and obesity.

Because the knee joint, sarcopenia, and the displacement of the body’s center of gravity are key to corporal balance, programs that combine exercise and diet may prevent falls in patients with obesity and KO. Further randomized controlled trials will be required in future research to test this. Sex and other variables, such as the quality of sleep, should also be considered.

## Figures and Tables

**Table 1:** Characteristics of selected studies by exercise intervention in patients with overweight or obesity suffering from degenerative knee osteoarthritis.

Study	Country	Group	Sample (*n*)	BMI	Treatment Length	Treatment Exercise Intervention	Main Results
De Rooij et al., 2017 [28]	Netherlands	Non-Intervention	42	35 ± 7.6	12 months	Individualized, comorbidity-adapted exercise program consisting of aerobic and strength training and training of daily activities. The control group received their current medical care for KO and were placed on a waiting list for exercise therapy.	At 3 months follow-up, the mean improvements in the intervention group were 33% on the WOMAC scale and 15% on the 6MWT. Tailored exercise therapy is efficacious in improving physical functioning in patients with KO and severe comorbidities.
		Intervention	60	36 ± 6.8			
Hsu et al., 2021 [29]	China	Non-Intervention	21	29.45 ± 2.59	12 months	An elastic band resistance exercise intervention was implemented. Each participant performed 10 repetitions/set of five sets/day of the aforementioned exercise movements 3 days a week for 12 weeks. Exercise intensity was increased by applying more force to the band to provide greater resistance or by switching to a thicker resistance band that created more resistance and thus increased exercise difficulty. A repetition maximum of 10 and rated perceived exertion of 13 (range of 6–20) were applied as the standards for the exercise program.	Individual diet control intervention combined with telemedicine-based resistance exercise intervention significantly improved the body composition, blood biochemistry, and lower-limb functional performance of the investigated population with comorbid conditions.
		Intervention	21	29.7 ± 2.64			
Lim et al., 2010 (A) [30]	Korea	Non-Intervention	20	27.86 ± 1.99	2 months	Each training session consisted of main activities in an aquatic gym for 30 min. The exercise program always started with 5 min of warm-up and ended with 5 min of cooldown. The exercise program was designed with 40 min duration per session, 3 times per week, for 8 weeks. Exercise intensity was maintained at the level of more than 65% of maximal heart rate by checking subject heart rates intermittently during exercise.	After the exercise intervention, BMI showed a small reduction in water exercise group. There was an enhancement in functional performance. Water exercise reduced the degree of activity interference by pain. Thus, water exercise may be an effective tool for patients with obesity who have difficulty in conventional exercises because of combined KO.
		Intervention	24	27.82 ± 1.56			
Lim et al., 2010 (B) [30]	Korea	Non-Intervention	20	27.86 ± 1.99	2 months	Participants assigned to the 8-week land-based exercise program underwent a generalized conditioning program also with knee specific exercises. Exercise duration was 40 min in each session, including 5 min of warm-up and 5 min of cooldown. The intensity of exercise began from 40% of the 1-repetition maximum for the beginner, but in advanced classes, 60% of 1-repetition maximum was applied, which is the usual intensity for geriatric patients. The exercises consisted of joint mobilization and strengthening exercises. Range-of-motion and stretching exercises of the hamstring, rectus femoris, tensor fascia latae, and calf muscles were included. Bicycling was also included for aerobic conditioning and fitness. A quadriceps isometric strengthening exercise was performed, along with other strengthening exercises, such as leg presses and leg extensions.	BMI showed a small reduction in exercise group. There was an enhancement in functional performance.
		Intervention	22	27.49 ± 1.66			
Messier et al., 2013 [26]	USA	Non-Intervention	52	33.7 ± 3.8	18 months	The exercise intervention was conducted for 1 h on 3 days/week. During the first 6 months, participation was center-based. After 6-month follow-up testing and a 2-week transition phase, participants could remain in the facility program, opt for a home-based program, or combine the two. The program consisted of aerobic walking (15 min), strength training (20 min), a second aerobic phase (15 min), and cool-down (10 min)	Compared with exercise participants, knee compressive forces were lower in diet participants, and IL-6 were lower in diet and diet + exercise participants. Pairwise between-group comparisons of WOMAC pain and function at 18 months revealed that the diet and exercise group had less pain relative to the exercise and diet groups. Pairwise between-group comparison showed that WOMAC function score was significantly better in the diet and exercise group relative to the exercise group.
		Intervention	52	33.6 ± 3.7			
							The 6MWT distance was 23.3 m farther in the diet and exercise group relative to the exercise group.
Rafiq et al., 2021 [31]	Malaysia	Non-Intervention	25	32.01 ± 3.89	1 month	Training sessions included strengthening exercises for the lower limbs in non-weight-bearing, sitting, or lying positions. Each training session started with 10 min of warm-up, 45–60 min of lower-limb resistance training, and 10 min of cooldown at the end of the training protocol. A cooldown period is essential after a training session and should last approximately 5–10 min. When static stretching is used as a part of warm-up immediately prior to exercise, it causes harm to muscle strength.	Short-term effects of the lower-limb rehabilitation protocol appear to reduce knee pain and stiffness only, but not physical function and BMI.
		Intervention	25	32.18 ± 4.49			
Schlenk et al., 2011 [27]	USA	Non-Intervention	13	33.3 ± 6	6 months	A fitness walking program was initiated in the fifth session (previous sessions consisted of a standardized educational program on sedentary lifestyles and obesity as risk factors for cardiovascular disease and KO) with the physical therapist to gradually progress subjects to fitness walking within their limitations, taking into account their symptoms. Subjects were to walk toward a goal of 150 min per week, but were permitted to distribute this time among multiple sessions as tolerated or preferred. The fitness walking program promoted performance of physical activity by graduated fitness walking goals, demonstration, and practice consistent with the self-efficacy strategy of mastery.	Results showed significant increases in self-reported performance of lower extremity exercise and participation in fitness walking, 6MWT distance, and Short Physical Performance Battery scores from baseline to 6-month follow-up with a trend of improvement in self-efficacy. Results suggest that the intervention was feasible, acceptable, and improved physical activity and function.
		Intervention	13	(Reported for both groups)			

6MWT, 6 min walk test; BMI, body mass index; KO, knee osteoarthritis; WOMAC, Western Ontario and McMaster Universities Osteoarthritis Index.

## Supplementary Materials

The following are available online at https://www.mdpi.com/article/10.3390/ijerph191710510/s1, Supplementary Material File S1: PRISMA Checklist; Supplementary Material File S2: Research strategy; Supplementary Material File S3: Sensitivity analysis.
