# Supplementary material for: Effectiveness of Exercise in Patients with Overweight or Obesity Suffering from Knee Osteoarthritis: A Systematic Review and Meta-Analysis
Source: Int J Environ Res Public Health. 2022 Aug 24;19(17):10510. doi: 10.3390/ijerph191710510 (PMC9518463; doi:10.3390/ijerph191710510)
Supplement: Supplementary file 1 [file ijerph-19-10510-s001.zip › Suplementary material File S2.pdf]

1. PubMed Field Tags: title & abstract

Date of Search: 2022-05-10
Publication years: 2002 to date
Number of Hits: 346

Search: **"knee osteoarthritis" AND exercise AND obesity**
"knee osteoarthritis"[All Fields] AND ("exercise"[MeSH Terms] OR "exercise"[All Fields] OR "exercises"[All Fields] OR "exercise therapy"[MeSH Terms] OR ("exercise"[All Fields] AND "therapy"[All Fields]) OR "exercise therapy"[All Fields] OR "exercise s"[All Fields] OR "exercised"[All Fields] OR "exerciser"[All Fields] OR "exercisers"[All Fields] OR "exercising"[All Fields]) AND ("obeses"[All Fields] OR "obesity"[MeSH Terms] OR "obesity"[All Fields] OR "obese"[All Fields] OR "obesities"[All Fields] OR "obesity s"[All Fields])
Translations
exercise: "exercise"[MeSH Terms] OR "exercise"[All Fields] OR "exercises"[All Fields] OR "exercise therapy"[MeSH Terms] OR ("exercise"[All Fields] AND "therapy"[All Fields]) OR "exercise therapy"[All Fields] OR "exercise's"[All Fields] OR "exercised"[All Fields] OR "exerciser"[All Fields] OR "exercisers"[All Fields] OR "exercising"[All Fields]
obesity: "obeses"[All Fields] OR "obesity"[MeSH Terms] OR "obesity"[All Fields] OR "obese"[All Fields] OR "obesities"[All Fields] OR "obesity's"[All Fields]

2. Web of Science Field Tags: TOPIC

Date of Search: Date of Search: 2022-05-10
Publication years: 2002 to date
Number of Hits: 368

3
"knee osteoarthritis" AND exercise AND obesity (All Fields): 368

2
"Osteoarthritis knee" AND exercise AND obesity (All Fields): 4

1
"Osteoarthritis knee“ AND "Exercise“ AND "Obesity" (All Fields): 4

3. ScienceDirect (SCOPUS) Field Tags: NO

Date of Search: Date of Search: 2022-05-10
Publication years: 2002 to date
Number of Hits: 458

TITLE-ABS-KEY ("knee osteoarthritis" AND exercise AND obesity)
