# Supplementary material for: Effectiveness of Exercise in Patients with Overweight or Obesity Suffering from Knee Osteoarthritis: A Systematic Review and Meta-Analysis
Source: Int J Environ Res Public Health. 2022 Aug 24;19(17):10510. doi: 10.3390/ijerph191710510 (PMC9518463; doi:10.3390/ijerph191710510)
Supplement: Supplementary file 1 [file ijerph-19-10510-s001.zip › Supplementary material File S3.pdf]

Supplementary material File S3.

*Sensitivity analysis*

| Study or Subgroup | Control Mean | Control SD | Control Total | Intervention Mean | Intervention SD | Intervention Total | Weight | Mean Difference IV, Random, 95% CI |
|---|---|---|---|---|---|---|---|---|
| de Rooij et al. 2017 | 8.6 | 3.6 | 63 | 6.6 | 3.6 | 63 | 17.9% | 2.00 [0.74, 3.26] |
| Lim et al. 2010 (A) | 4.55 | 1.88 | 20 | 3.27 | 1.67 | 24 | 25.1% | 1.28 [0.22, 2.34] |
| Lim et al. 2010 (B) | 4.55 | 1.88 | 20 | 3.46 | 1.3 | 22 | 29.0% | 1.09 [0.10, 2.08] |
| Rafiq et al. 2021 | 9.52 | 2.08 | 25 | 8.85 | 1.49 | 25 | 28.1% | 0.67 [−0.33, 1.67] |
| **Total (95% CI)** | | | **128** | | | **134** | **100.0%** | **1.18 [0.65, 1.71]** |

Heterogeneity: $Tau^2 = 0.00$; $Chi^2 = 2.69$, df = 3 (P = 0.44); $I^2 = 0\%$
Test for overall effect: Z = 4.36 (P < 0.0001)

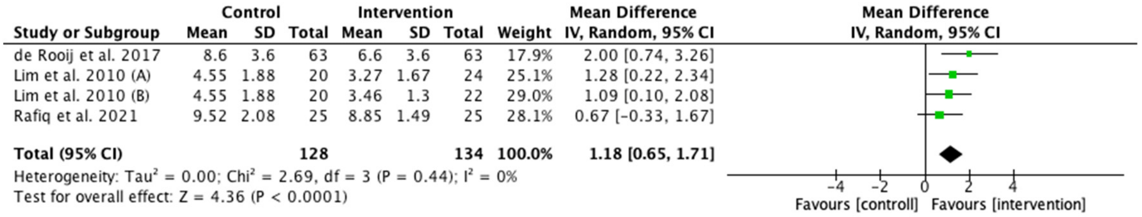


**Figure S1**. Sensitivity analysis excluding studies [26,29] comparing a 'diet and exercise' group versus a 'diet' group. Effects interventions on pain. CI, confidence interval.

Note: lower scores are indicating lower levels of pain.

| Study or Subgroup | Control Mean | Control SD | Control Total | Intervention Mean | Intervention SD | Intervention Total | Weight | Mean Difference IV, Random, 95% CI |
|---|---|---|---|---|---|---|---|---|
| de Rooij et al. 2017 | 31.4 | 4.23 | 63 | 23.5 | 13.4 | 63 | 59.2% | 7.90 [4.43, 11.37] |
| Lim et al. 2010 (A) | 27.6 | 18.3 | 24 | 20.9 | 9.9 | 20 | 9.8% | 6.70 [−1.81, 15.21] |
| Lim et al. 2010 (B) | 27.6 | 18.3 | 22 | 23.6 | 12.8 | 20 | 7.9% | 4.00 [−5.48, 13.48] |
| Rafiq et al. 2021 | 23.9 | 12.37 | 25 | 22.07 | 11 | 25 | 16.9% | 1.83 [−4.66, 8.32] |
| Schlenk et al. 2011 | 22.9 | 14.9 | 13 | 17.3 | 13.1 | 13 | 6.1% | 5.60 [−5.18, 16.38] |
| **Total (95% CI)** | | | **147** | | | **141** | **100.0%** | **6.30 [3.64, 8.97]** |

Heterogeneity: $Tau^2 = 0.00$; $Chi^2 = 2.89$, df = 4 (P = 0.58); $I^2 = 0\%$
Test for overall effect: Z = 4.63 (P < 0.00001)

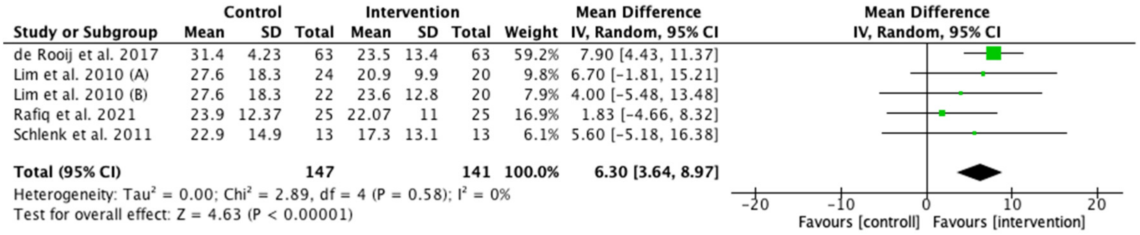


**Figure S2**. Sensitivity analysis excluding studies [26,29] comparing a 'diet and exercise' group versus a 'diet' group. Effects interventions on physical function. CI, confidence interval.

Note: lower scores indicating lower levels of symptoms or physical disability.

| Study or Subgroup | Intervention Mean [meters] | Intervention SD [meters] | Intervention Total | Control Mean [meters] | Control SD [meters] | Control Total | Weight | Mean Difference IV, Random, 95% CI |
|---|---|---|---|---|---|---|---|---|
| de Rooij et al. 2017 | 465.3 | 93.9 | 63 | 423 | 114.8 | 63 | 80.0% | 42.30 [5.68, 78.92] |
| Schlenk et al. 2011 | 426.2 | 92.96 | 13 | 368.78 | 97.47 | 13 | 20.0% | 57.42 [−15.80, 130.64] |
| **Total (95% CI)** | | | **76** | | | **76** | **100.0%** | **45.33 [12.57, 78.08]** |

Heterogeneity: $Tau^2 = 0.00$; $Chi^2 = 0.13$, df = 1 (P = 0.72); $I^2 = 0\%$
Test for overall effect: Z = 2.71 (P = 0.007)

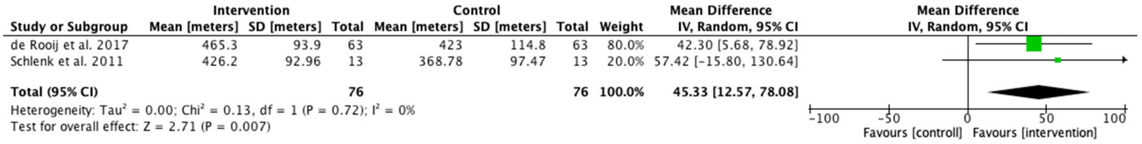


**Figure S3.** Sensitivity analysis excluding studies [26,29] comparing a 'diet and exercise' group versus a 'diet' group**.** Effects interventions on distance (meters) reached in the 6-minute test walk. CI, confidence interval.
